# The nonlinear relationship between psychological contract and burnout among Chinese government employees: a moderated mediation model

**DOI:** 10.3389/fpubh.2025.1729716

**Published:** 2026-01-12

**Authors:** Yurong Wu, Weihu Liang

**Affiliations:** 1 School of Public Policy and Management, Guangxi University, Nanning, China; 2 Guangdong Industry Polytechinc Students Affair, Guangdong Industry Polytechinc University, Guangzhou, China

**Keywords:** bianzhi, burnout, expectancy disconfirmation, perceived insider status, psychological contract, self-regulation

## Abstract

**Background:**

Grassroots government employees of the Chinese government are crucial for realizing Chinese-style modernization. However, the mismatch between governance resources and demands causes high burnout among them, impacting work efficiency and effectiveness. This study, based on expectancy disconfirmation theory (EXT) and self-regulation theory (SRT), constructs a moderated mediation model to show how psychological contracts (PC), perceived insider status (PIS), and bianzhi affect the burnout.

**Objective:**

This study aims to explore the nonlinear relationship between PC and burnout among Chinese grassroots government employees, and reveal the mediating role of PIS and the moderating role of bianzhi.

**Methods:**

A total of 527 Chinese government employees was used. Data were analyzed using the stepwise regression method, the MEDCURVE plugin and the PROCESS macro bootstrap approach.

**Findings and conclusion:**

The results illustrated that there is an inverted U-shaped relationship between PC and burnout. PC has an inverted U-shaped mediating effect via PIS. Bianzhi moderates both of them, and the mediating effect of PIS is affected by it.

**Implications:**

This research findings are supported by the relevant conclusions of EXT and SRT, providing evidence for theory development. It also comprehensively explains relationships among PC, PIS, bianzhi and burnout. This study provides theoretical support and practical references for alleviating burnout of grassroots government employees and optimizing human resource management in public sectors. It innovatively verifies the inverted U-shaped relationship between PC and burnout of grassroots government employees, clarifies the moderating mechanism of bianzhi differences on the mediating path, and enriches the research perspective of occupational health in public sectors.

## 1 Introduction

Since the 18th National Congress of the Communist Party of China (1), president Xi Jinping has delivered a series of crucial speeches regarding grassroots governance, elucidating its significant status and role within the national governance system and emphasizing that the modernization of grassroots governance constitutes the cornerstone and guarantee for realizing Chinese-style modernization. The grassroots government, serving as a crucial bridge between the state, society, and the masses, leads to grassroots governments and their employees frequently undertaking a large number of affairs (2). Grassroots government employees, who are public servants engaged in grassroots government work and positioned at the forefront of grassroots operations, directly interact with the masses. Their primary task lies in resolving the issues concerning the livelihoods of the masses, such as food, clothing, housing, transportation, employment, education, social security, and medical care. As the essential protagonists in grassroots governance, their work attitudes and psychological states play a pivotal role. Not only do these factors have a direct bearing on the quality and efficacy of grassroots operations, but they also exert a profound impact on the public trustworthiness of the Party and the government, and are closely intertwined with the success of policy implementation and the overall effectiveness of national governance.

With the continuous advancement of Chinese-style modernization, grassroots governance work has become increasingly arduous and intricate. The mismatch between governance resources and governance tasks has become more prominent, leading to the prominent problem of burnout among grassroots government employees (3). To a certain extent, this has hindered the process of Chinese-style modernization. Therefore, enhancing the work incentive of grassroots government employees and the efficacy of grassroots governance is of crucial importance for promoting the high-quality development of grassroots governance. Subsequently, how to alleviate the burnout of grassroots government employees has emerged as a prominent topic of common concern in both the academic community and society.

Most existing studies explore the relationship between psychological contract (PC) and burnout based on the social exchange theory (SET), conservation of resources theory (COR) and Job Demands-Resources model (JD-R). Generally, it is posited that burnout is the consequence of psychological contract breach (PCB) between individuals and organizations, and the two are considered to have a linear relationship. PC is regarded as an explicit or implicit promise formed by individuals in the interaction process with the organization, reflecting the expectations of individuals for the reciprocal commitment between their obligations (i.e., what they do for the organization) and rights (i.e., what return they expect) (4, 5). SET points out that individual behavior and attitude are a manifestation of social exchange. Based on the principle of reciprocity, they decide whether to continue the exchange behavior according to the perceived input and return (6). Therefore, PC is regarded as the criterion for individuals to evaluate whether the balance between their input and return is achieved (7). However, when they think that the input and return are unequal and PCs are broken, they often respond with negative attitudes and behaviors. At the same time, COR believes that individuals have the tendency to obtain, maintain and cultivate their own resources (8). When they think that their potential or actual resources are lost, burnout will appear (9). This, in turn, causes the resource loss cycle, where employees experience continuous resource depletion and face difficulties in achieving recovery, ultimately affecting their work engagement (10). Individuals usually regard PCB as the loss of valuable resources (11). Therefore, based on COR, burnout is considered as the direct reaction of individual PCB.

On this basis, some scholars further propose JD-R, which classifies all factors related to work into two broad categories: job demands and job resources. Specifically, in a high-demand but low-resource work environment, employees’ burnout will significantly increase, because the depletion of health and motivation caused by the inequality between work resources and demands. In contrast, in an environment with abundant work resources, employees often exhibit high work motivation, and their work engagement is notably enhanced (12, 13). In essence, psychological contract fulfillment (PCF) contains the care and encouragement of the organization for employees. It is regarded as a resource that employees expect to obtain in the exchange relationship with the organization and is an important form of job resources. That is to say, PCF will improve individuals’ engagement and well-being, and reduce burnout (14–16).

However, existing studies have notable limitations. Firstly, the understanding of the relationship between PC and burnout is rigid. The most studies overlook the proposition in COR that “the negative impact of resource loss on individuals may be far more significant than the positive impact of acquiring similar resources” and fail to recognize that the effects of varying degrees of PCF may be nonlinear. Second, most existing studies focus on groups such as nurses (17), teachers (18), and police (19), with insufficient attention paid to grassroots government employees, particularly the lack of targeted research on this group under the dual structure based on bianzhi. Third, existing studies have not fully clarified the intermediate transmission mechanism through which PC influences burnout, nor have they incorporated bianzhi, a unique institutional variable in Chinese grassroots governance, thereby making it difficult to explain the mechanism driving burnout differences among grassroots government employees with different identities.

Based on these research gaps, this study draws on expectancy disconfirmation theory (EDT) and self-regulation theory (SRT) to construct an analytical framework of “PC- perceived insider status (PIS)- burnout.” It specifically introduces bianzhi as a moderating variable to analyze the nonlinear relationship between PC and burnout among Chinese grassroots government employees. The marginal contributions of this study are summarized as follows: Firstly, it breaks through the linear cognitive framework and empirically verifies the nonlinear relationship between PC and burnout of Chinese grassroots government employees, indicating that the degree to which PCF alleviates employees’ burnout is not constant. Secondly, it explores the nonlinear mediating mechanism and clarifies the inverted U-shaped mediating role of PIS in the relationship between PC and burnout. Thirdly, it highlights Chinese contextual characteristics by systematically verifying, for the first time, the moderating effect of bianzhi on the path “PC → PIS → burnout,” thus explaining the differential mechanisms between biannei personnel (possess formal bianzhi) and bianwai personnel (without formal bianzhi). These research findings not only enrich the theoretical system of burnout and PC but also provide new insights for grassroots governments to accurately alleviate burnout among personnel with different identities and optimize human resource management.

## 2 Theoretical basis and research hypotheses

### 2.1 The nonlinear relationship between psychological contract and burnout

PC was first proposed by American behaviorist Chris Argyris and introduced into the management field in the 1960s. It refers to the informal and unwritten contract between employees and organizations. PC can be roughly divided into two dimensions: transactional contract and relational contract (20). The former regards the relationship between individuals and organizations as a short-term transactional relationship based on economic exchange, while the latter regards the relationship between the two as a long-term exchange relationship based on emotional exchange (21). In addition, Herriot et al. (22) believes that PC is the perception of what responsibilities each other should provide for based on formal or informal agreements in the employment relationship between organizations and individuals. Therefore, according to the differences in responsible subjects, PC can be roughly divided into two categories: employee PC and organizational PC (23). It should be noted that since the subject of organizational PC is difficult to determine, this study focuses on employee PC, that is, PC of grassroots government employees.

Burnout, also known as “job burnout,” is an important concept in the field of psychology. It was first proposed by Freudenberger to describe the psychological exhaustion phenomenon exhibited by individuals in the medical and service industries due to excessive demands on energy, strength or resources at work (24). Maslach (25) believes that job burnout is a continuous process, consisting of stages such as loss of work enthusiasm and vitality, negative work attitude, and negative evaluation of value and meaning. It is a comprehensive manifestation of emotional exhaustion, depersonalization and reduced personal accomplishment of individuals under long-term work pressure. Based on this, Maslach Burnout Inventory (MBI) is compiled with these as the main dimensions, providing a theoretical model for measuring the degree of individual job burnout and is widely recognized in the academic community (25).

EDT holds that the direction and magnitude of the gap between consumers’ pre-consumption expectations and their actual post-consumption perception determine whether they are satisfied, as well as the degree of that satisfaction. When actual perception exceeds expectation, positive disconfirmation is formed (leading to satisfaction), conversely, negative disconfirmation is formed (leading to dissatisfaction) (26, 27). The core of EDT is that the formation of customer satisfaction is not simply based on the absolute magnitude of performance perception, but takes customer expectation as the reference point for evaluation and judgment (28), and it is widely used in the field of consumer behavior. Like customer satisfaction, burnout is a comprehensive reaction characterized by physical and mental exhaustion formed, when individuals perceive the work requirements and personal investment do not match organizational returns, job burnout then forms (29). It is the attitude and behavioral reaction after individuals compare subjective evaluation and psychological cognition, is also affected by individuals’ expectations and actual feelings of organizational fulfillment of commitments or responsibilities. At the same time, PCB and PCF are the subjective perception of individuals on whether the organization fulfills the commitments or responsibilities in the contract (30). COR points out that the degree of influence of resource depletion and resource acquisition is not completely consistent. Combined with this, it can be inferred that burnout is jointly affected by individuals’ expectations of the organization and PC (actual perception), and there may also be a nonlinear relationship between PC and burnout. Meanwhile, SRT think that individuals will adjust their behavioral responses according to the gap between reality and ideal to narrow the difference (31). Based on these, this study hypothesizes that when grassroots government employees’ actual perception derived from PC are close to or exceeds their expectations, positive disconfirmation is formed, and individual burnout will be at a relatively low level. Conversely, if there is a significant gap between grassroots government employees’ actual perceptions derived from the PC and their expectations, they are highly likely to exhibit a strong negative reaction, and the degree of their burnout will significantly increase. Therefore, this study proposes the following hypothesis:

*H1*: There is an inverted U-shaped relationship between PC and burnout. Specifically, the degree of PCF generally mitigates burnout, while a significant gap between grassroot government employees’ actual perceptions (that is PC) and their individual expectations leads to a marked increase in burnout.

### 2.2 The mediating role of perceived insider status

PIS refers to an individual’s perception of the personal space and acceptance they obtain as an organizational member, which is usually formed by the combination of socialization practices and common interests (32, 33). In the process of organizational socialization, PIS emerges with the establishment of PC and increases accordingly (34). Meanwhile, when the organization fulfills its promised obligations and responsibilities (35), it satisfies the economic resources and social emotional resources expected by individuals (16). This may strengthen employees’ sense of organizational identity and belonging, prompting them to make contributions to repay the organization (36).

PIS is like to the concept of Chinese “collectivist culture” (37). In Chinese local organizations, individuals are accustomed to informally demarcating insiders and outsiders (38). When they consider themselves as insiders, they often have a stronger sense of belonging and dependence on the organization, also are more willing to take the initiative to assume responsibilities (39). Base on this, it can be though that individuals with high PIS usually have a lower level of job burnout.

However, by integrating EDT and SRT, it can be inferred that the impact of PIS is not always positive, and a nonlinear relationship may also exist between PIS and burnout. Specifically, although grassroots government employees’ perceived organizational support and recognition increase as their PIS strengthens, the depletion of psychological resources is often difficult to supplement and restore in a timely manner (40). When grassroots government employees hold high expectations, improvements in PIS may not necessarily reduce their burnout. Instead, the significant gap between their actual perceptions and expectations may lead to negative disconfirmation. Under such circumstances, grassroots government employees’ job burnout levels increase as their PIS strengthens. In addition, SRT points out that individual behavior choices change with changes in their own cognition. Therefore, PC and PIS, as personal cognitive evaluations of the organization, are very likely to trigger a series of changes in ideas and behaviors related to work. Combined with the above analysis, this research argues that PIS is likely to play a mediating role in the relationship between PC and burnout. Therefore, this study puts forward the following hypothesis:

*H2*: PC has a positive effect on PIS.

*H3*: There is an inverted U-shaped relationship between PIS and burnout. Specifically, PIS mitigates burnout, but a significant gap between grassroots employees’ actual perceptions and their individual expectations leads to a marked increase in burnout levels.

*H4*: PC has an inverted U-shaped mediating effect on burnout through PIS.

### 2.3 The moderating role of bianzhi

Bianzhi is a fundamental institution with distinct Chinese characteristics. It refers to a system that defines government functions, the number of institutions, and the number of employees hired by each institution (41). It exists in all core bureaus (jiguan danwei) and extra-bureaucracies (shiye danwei). These two categories operate in parallel within China’s bureaucratic system, which differs from Western democratic systems in its dual hierarchical structure of the Party and the government. Specifically, core bureaus are mainly responsible for political, administrative, and regulatory work. The extra-bureaucracies, which are public welfare institutions affiliated with core bureaus, are primarily in charge of a series of delegated tasks.

The function of the bianzhi system is to offer regulatory guidelines regarding the quantity of public employees and the magnitude of public budgets, thereby furnishing a foundation for ascertaining the optimal scale of each public entity (42). In a narrow sense, the current types of bianzhi in China are mainly divided into two categories: administrative bianzhi (xingzheng bianzhi) and subsidiary bianzhi (shiye bianzhi). The public employees with xingzheng bianzhi are called for civil servants and public employees with shiye bianzhi are called for shiye personnel. In practice, although civil servants and shiye personnel are managed according to different salary and promotion hierarchies, both possess formal bianzhi (formally established posts).

However, in the current scenario of grassroots governance in China, there exists a prominent contradiction. The number of positions with bianzhi is fixed and cannot be augmented, whereas the workload of grassroots governance is constantly rising. The core issue is that existing bianzhi standards are highly rigid and difficult to modify. This makes it challenging for them to align with the growing and evolving demands faced by local government. Given the aforesaid predicament, a large number of grassroots governments have chosen to engage in the practice of recruiting personnel in excess of the stipulated quota (chaobian), with the aim of mitigating the shortfall in the quantity of grassroots government employees (43). The over-quota employees (chaobian personnel) are called bianwai personnel. Their salaries are paid by the grassroots governments that employ them. They do not possess formal bianzhi and similar to “contract agents” in the EU government, whose employment can be terminated at any time. To better control the increasing number of bianwai personnel, in recent years, the Chinese central government has issued a requirement of strictly controlling the number of bianwai personnel. This means that bianwai personnel have also become the object of bianzhi management. Therefore, in a broad sense, the types of bianzhi structure in China mainly involve three categories: xingzheng bianzhi, shiye bianzhi, and bianwai bianzhi (44). Based on whether they hold formal bianzhi (formally established posts), this study divides grassroots government employees into two categories: biannei personnel (holding xingzheng bianzhi or shiye bianzhi, i.e., with formal bianzhi) and bianwai personnel (holding bianwai bianzhi, i.e., without formal bianzhi). This classification is adopted to explore the moderating role of bianzhi in PC and burnout.

Existing research shows that there are significant differences in PIS between contractual, temporary or marginal employees and fulltime employees (45). Undeniably, there is an obvious “bianzhi segregation” phenomenon in China. Bianwai personnel and biannei personnel still show a relatively severe asymmetric pattern in terms of scope of rights and responsibilities, salary and welfare, promotion and development, social security and even social status (44). The sense of unfairness cause bianwai personnel to lack of sufficient work enthusiasm and initiative (46), and the sense of value and belonging to the profession also decreases, giving rise to negative burnout emotions (47). In other words, PIS of grassroots government employees is not only influenced by the degree of PCF but also by their bainzhi, which may moderate the effect of PC on PIS.

**Figure 1:**
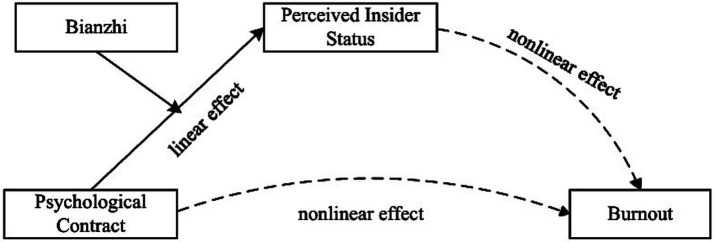
Theoretical framework chart.

By contrast, there is a stronger linear relationship between the degree of PC and PIS among grassroots bianwai personnel. Theoretically, their PIS plays a stronger mediating role in the relationship between PC and burnout. However, in practice, most bianwai personnel perceive themselves as “marginalized individuals” within organizations. In recent years, Chinese grassroots governments have continuously standardized the management system for bianwai personnel. They have also provided these personnel with stable expectations and recognized their legitimate status through semi-formal management mechanisms such as implicit political incentives, differentiated economic incentives, and auxiliary incentives. However, this initiative has not fundamentally altered the identity attribute of bianwai personnel, nor has it eliminated the institutional exclusion they face (48). In essence, individuals tend to define themselves through their identities. Their identity cognition not only affects their sense of belonging to the profession, but also affects their work attitudes and performance in the organization (49). Drawing on SRT, which posits that “individuals adjust their behavioral strategies based on the discrepancy between actual perceptions and expectations” (31), this study can be inferred that the mediating effect of PC on burnout through PIS is moderated by bianzhi, and this mediating effect is stronger among biannei personnel. Based on the above analysis, this paper puts forward the following hypotheses (Figure 1):

*H5*: Bianzhi plays a moderating role in the relationship between PC and PIS.

*H6*: The nonlinear mediating mechanism of PC affecting burnout through PIS is moderated by bianzhi. Specifically, the mediating effect of PIS is stronger among biannei personnel than among their bianwai personnel.

## 3 Materials and methods

### 3.1 Participants and procedures

This study takes grassroots government employees in City A, Province G, China as the object and obtains relevant data by filling out anonymous questionnaires. To ensure the representativeness of the sample, this study adopted a combination of multistage stratified sampling and simple random sampling to determine the survey respondents. In the first stage, with City A of Province G as the research area, stratification was conducted according to the administrative levels of district/county-level governments and sub-district/township-level governments to avoid sample bias caused by a single administrative level. In the second stage, within each administrative level, further stratification was carried out based on the institutional nature of core bureaus and extra-bureaucracies, and the sample proportion of units with different natures was ensured to be consistent with the overall structure. In the third stage, simple random sampling was used to randomly select 4–5 specific units from each stratified category, and finally 18 grassroots government units were identified as the survey subjects.

From June to August 2024, after obtaining the informed consent of the respondents, this study adopted a combination of online and offline methods to conduct a questionnaire survey on all government employees in the selected grassroots units. A total of 550 questionnaires were distributed in this survey. After excluding invalid questionnaires with missing answers to important questions, obvious logical errors, and excessively short response time, 527 valid questionnaires were obtained, with an effective rate of 95.81%. According to statistical standards, the sample size is usually required to be 5–10 times the number of measurement items (50). This study included 27 measurement items, and the number of valid questionnaires was 527. The effective sample size was 19.52 times the number of measurement items, which meets the general statistical requirements. The demographic distribution of the sample is shown in Table 1.

**Table 1:** Results of descriptive statistics for the sample (*N* = 527).

Category		Frequency
Gender	Female	220
	Male	307
Age	18–30 years	62
	31–40 years	136
	41–50 years	189
	51–60 years	140
Educational level	High school and below	19
	Junior college	98
	Bachelor	367
	Master and Doctor	43
Bianzhi categories	Binawai personnel	102
	Biannei personnel	419
Administrative rank	Bianwai personnel	108
	Clerk	233
	Section-level cadres	158
	Division-level cadres	28
Years of work	10 years and below	243
	11–20 years	102
	21–30 years	115
	30 years and above	67

### 3.2 Model construction and interpretation

Based on the foregoing analysis, this study mainly examines nonlinear relationships, nonlinear mediating effects, moderating effects, and moderated mediating effects. Therefore, the following model is constructed with reference to the testing methods recommended by Baron and Kenny (51), Preacher and Hayes (52), Faber and Walter (53), etc.

$$Y=\alpha_{0}+\alpha_{1}X+\alpha_{2}X^{2}+\alpha_{3}\text{controls}+\varepsilon$$ (1)

$$M=\beta_{0}+\beta_{1}X+\beta_{2}\text{controls}+\varepsilon$$ (2)

$$\begin{array}{c} Y=\gamma_{0}+\gamma_{1}M+\gamma_{2}M^{2}+\gamma_{3}\text{controls}+\varepsilon \end{array}$$ (3)

$$\begin{array}{c} Y=\eta_{0}+\eta_{1}X+\eta_{2}M+\eta_{3}M^{2}+\eta_{4}\text{controls}+\varepsilon \end{array}$$ (4)

$$\begin{array}{c} \theta =(\frac{\partial M}{\partial X})(\frac{\partial Y}{\partial M})=\beta_{1}(\eta_{1}+2\eta_{2}M) \end{array}$$ (5)

Equation (1) examines the inverted U-shaped relationship between PC and burnout of grassroots personnel. Equation (2) examines the linear relationship between PC and PIS. Equation (3) examines the inverted U-shaped relationship between PIS and burnout. Equations (4) and (5) examine the nonlinear mediating role of PIS between PC and burnout. In Equation (5), *θ* is used to estimate the ratio of the change in the dependent variable (Y) indirectly affected by the independent variable (X) through the mediating variable (M). Among them, X is PC, Y is burnout, M is PIS, Z is bianzhi, controls are the general term for control variables, *α*, *β*, *γ*, *η*, *δ* are regression coefficients of each variable, and *ε* is a random disturbance term.

### 3.3 Variable design

The variables in this study include burnout, PC, PIS and bianzhi. In terms of measuring each variable, mature scales with good reliability and validity which in existing research are all referred to. The survey respondents judge the descriptions in the questionnaire items according to their own actual feelings and experiences.

#### 3.3.1 Dependent variable

The dependent variable of this study is burnout. This study uses the burnout scale (MBI-Chinese version) more suitable for the Chinese context revised by Li and Shi (54) based on MBI. And it is used to measure the degree of burnout of Chinese grassroots government employees from three dimensions of emotional exhaustion, cynicism, and professional efficacy. The questionnaire is evaluated by using Likert’s seven-point scoring method. The higher the value, the higher the degree of burnout. The results of reliability and validity tests show that the Cronbach’s *α* coefficient of the overall questionnaire is 0.81 and the KMO value is 0.83, indicating that the internal consistency of this questionnaire is relatively high.

#### 3.3.2 Independent variable

The independent variable of this study is PC. This study measures PC of Chinese grassroots government employees according to the questionnaire on PC of Chinese civil servants which designed by Zhang (55), p. 18, it mainly classifies from two aspects of transactional contract and relational contract to measure. It is evaluated by using Likert’s five-point scoring method. The higher the score, the higher the degree of PCF. The Cronbach’s α coefficient of this questionnaire is 0.92 and the KMO value is 0.83, it means that the questionnaire has a high internal consistency.

#### 3.3.3 Mediating variable

The mediating variable of this study is PIS. The measurement scale for PIS is modified based on the instrument development by Stamper and Masterson (33), combined with the results of semi-structured interviews of Chinese grassroots government employees. This modification makes the scale more suitable for the Chinese context. The scale adopts a seven-point Likert scoring method, where higher scores indicate a higher level of PIS. The Cronbach’s α coefficient of this questionnaire is 0.75 and the KMO value is 0.71, indicating good internal consistency and validity.

#### 3.3.4 Moderating variable

The moderating variable of this study is bianzhi. In this study, the bianzhi type of grassroots employee is determined by the question “Do you have a formal bianzhi?.” Specifically, bianwai personnel are coded as “0,” and biannei personnel are coded as “1.”

#### 3.3.5 Control variable

Drawing on previous research findings, this study includes the following control variables to ensure the accuracy of the analysis results: gender (0 = female, 1 = male), age (1 = 18-30 years, 2 = 31-40 years, 3 = 41-50 years, 4 = 51-60 years), educational level (1 = high school and below, 2 = junior college, 3 = bachelor, 4 = master and doctor), administrative rank (1 = bianwai personnel, 2 = clerk, 3 = section-level cadres, 4 = division-level cadres), and years of work (1 = 10 years and below, 2 = 11-20 years, 3 = 21-30 years, 4 = 30 years and above). These variables are included because they may influence the study’s outcomes and are characteristic of grassroots government employees.

### 3.4 Statistical analysis

In this study, SPSS 26.0 was used to conduct descriptive statistical analysis and correlation analysis on the dataset. The stepwise regression technique was adopted to dissect the mediating effect of PIS and the moderating effect of bianzhi. Meanwhile, the MEDCURVE plug-in and the PROCESS macro bootstrapping were applied to test the significance of the nonlinear mediating effect and moderating effect. During the analysis process, all variables were standardized, and control variables including gender, age, educational level, administrative rank, and years of work of grassroots government employees were incorporated to control for potential confounding effects.

## 4 Results

### 4.1 Descriptive statistics and correlation analysis

The results of the correlation analysis of each variable are shown in Table 2. The results show that there are no obvious abnormalities in the descriptive statistics and correlation coefficients of each variable. Among them, PC (*r* = 0.399, *p* < 0.001) is significantly positively correlated with PIS. PC (*r* = −0.142, *p* < 0.01) and PIS (*r* = −0.233, *p* < 0.001) are both significantly negatively correlated with burnout. In addition, in order to eliminate the multicollinearity problem caused by the high correlation between the first-order term and its square term, this paper uses the method of centralizing all variables involving square terms to centralize PC and PIS. After data processing, the variance inflation factor (VIF) of the variables involved in the model is less than 5, so there is no multicollinearity problem.

**Table 2:** Descriptive statistics and correlation analysis results.

Variable	Mean	Sd	1	2	3	4	5	6	7	8
1 Gender	1.42	0.49								
2 Age	2.78	0.97	−0.285^***^							
3 Educational level	2.82	0.62	0.024	−0.260^***^						
4 Administrative rank	2.22	0.81	−0.235^***^	0.459^***^	0.160^***^					
5 Years of work	2.01	1.09	−0.076	0.612***	−0.327^***^	0.196^***^				
6 Bianzhi	0.80	0.40	−0.199^***^	0.462***	0.053	0.741^***^	0.294^***^			
7 PC	2.27	0.95	−0.061	0.067	−0.064	0.162^***^	0.053	0.161^***^		
8 PIS	5.84	1.11	−0.039	0.178^***^	−0.061	0.241^***^	0.134^**^	0.269^***^	0.399^***^	
9 Burnout	3.56	1.08	−0.104*	0.002	−0.073	−0.103^*^	0.020	−0.099^*^	−0.142^**^	−0.233^***^

*N* = 527; ***p < 0.001, **p < 0.01, *p < 0.05. PC, Psychological contract; PIS, Perceived insider status.

### 4.2 Net effect of psychological contract on burnout grassroots government employees

According to the results of M1, M2, and M3 in Table 3, both the first-order term (*β* = −0.215, *p* < 0.001) and the second-order term of PC (*β* = −0.186, *p* < 0.001) significantly affect burnout of grassroots government employees. The regression coefficients are significantly negative. Compared with M2, the change in the coefficient of determination (Δ*R*^2^) of M3 also reaches a significant level. This result indicates that there is a nonlinear inverted U-shaped relationship between PC and burnout, and H1 is proved. Further, this study uses the formula “Z_X_ = -b/2a” proposed by Cohen to calculate the zero-slope point of the curve (56), pp. 195–201. Among them, the variable “Z_X_” denotes the independent variable subsequent to the process of centralization, “a” represents the unstandardized regression coefficient corresponding to the quadratic term, and “b” signifies the unstandardized regression coefficient pertaining to the linear term.

**Table 3:** The results of regression analysis (*N* = 527).

Predictors	Dependent variable							
	Burnout				PIS			
	M1	M2	M3	M6	M7	M4	M5	M8
Independent variable								
PC		−0.137^**^	−0.215^***^		−0.092		0.367^***^	0.514^***^
PC^2^			−0.186^***^		−0.093			
Mediating variable								
PIS				−0.416^***^	−0.321^***^			
PIS^2^				−0.261^***^	−0.205^***^			
Moderating variable								
Bianzhi								0.125^*^
Interaction								
PC × Bianzhi								−0.110^*^
Control variables								
Gender	−0.132^**^	−0.136^**^	−0.134^**^	−0.111^*^	−0.115^*^	0.030	0.041	0.047
Age	0.001^**^	−0.009	−0.049	0.012	−0.012	0.029	0.057	0.044
Educational level	−0.042	−0.057	−0.064	−0.057	−0.064	−0.077	−0.038	−0.028
Administrative rank	−0.132^*^	−0.104^*^	−0.083	−0.071	−0.060	0.238	0.163	0.069
Years of work	0.021	0.024	0.043	0.037	0.045	0.047	0.038	0.025
*R* ^2^	0.031	0.049	0.077	0.113	0.121	0.072	0.202	0.217
△*R*^2^	0.021	0.038	0.064	0.101	0.106	0.063	0.193	0.205
*F*	3.300	4.450^***^	6.170^***^	9.486	8.259^***^	8.114	21.916^***^	17.995^***^

***p < 0.001, **p < 0.01, *p < 0.05. PC, Psychological contract; PIS, Perceived insider status. The coefficients in the table are stand-ardized regression coefficients.

**Figure 2:**
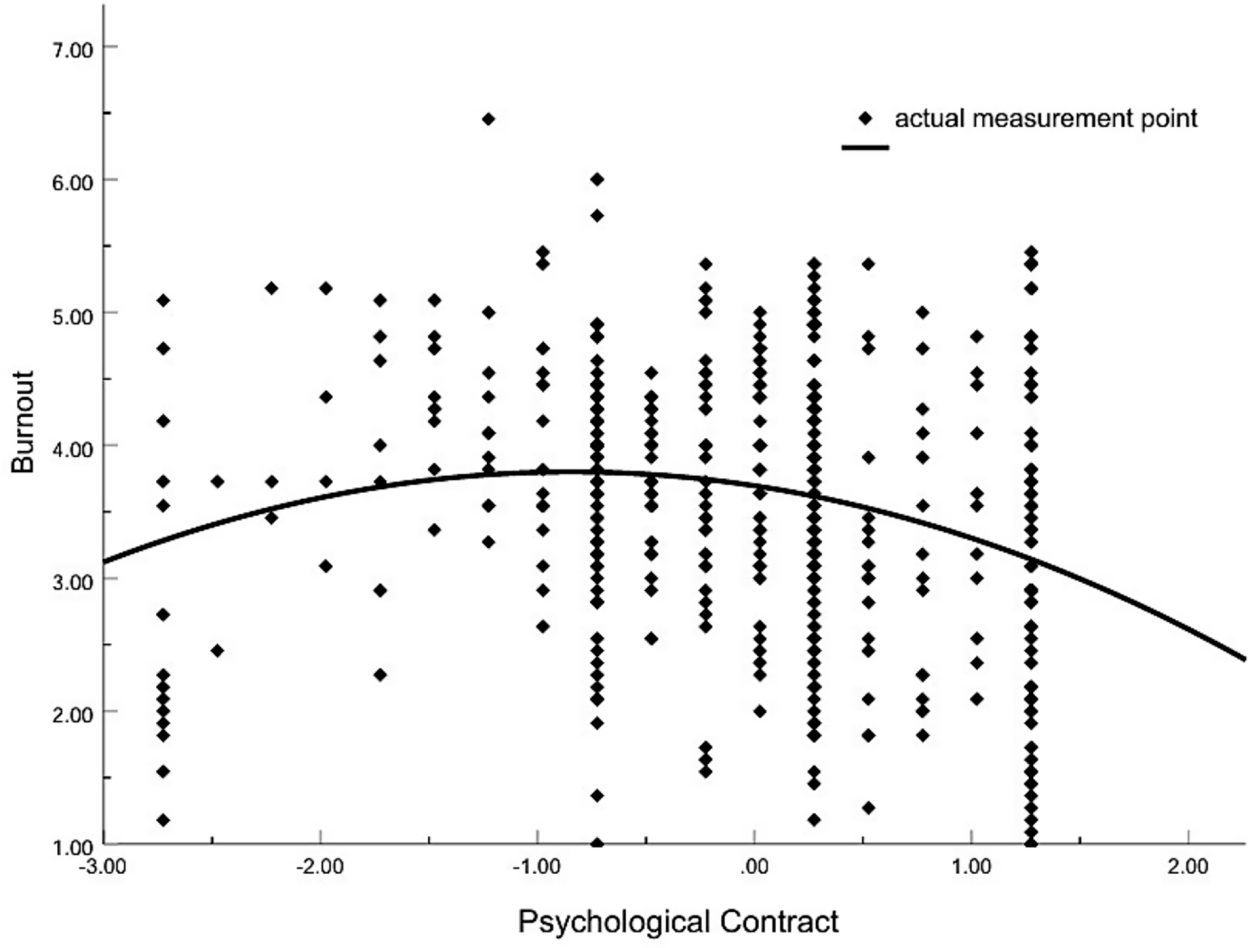
The inverted U-shaped relationship between PC and burnout.

The results show that the zero-slope point of the inverted U-shaped curve is (−0.920, 4.835). The inverted U-shaped relationship between PC and burnout is shown in Figure 2. For Chinese grassroots government employees, the relationship between PC and burnout is initially positive. When the level of PC reaches the threshold, grassroots government employees’ burnout is maximized. Beyond this threshold, however, the two variables are negatively correlated, burnout decreases as PC increases. While this finding may seem to contradict the traditional assumption that “higher PC is associated with lower burnout,” it is actually logically consistent. Van Ryzin (57) argues that “when evaluating public services, individuals do not rely solely on the actual quality of services experienced; they also refer to the discrepancy between actual quality and prior expectations.” This logic is equally applicable to grassroots government employees’ perception of PC. Their judgment of PC is essentially a comparison between actual perceptions and personal expectations. Thus, when PC is low and employees’ expectations of the organization are similarly low, they tend to disengage emotionally and cognitively from their work. Operating only in a passive state of “meeting minimal job requirements,” they avoid the frustration of “unmet effort” and the emotional drain of “holding expectations for the organization,” resulting in a relatively low level of burnout.

### 4.3 The mediating effect of perceived insider status

This study uses the mediation effect test method proposed by Baron and Kenny (51) to verify the mediating role of PIS. This method mainly consists of three steps: (1) testing whether the independent variable significantly affects the mediating variable; (2) testing whether the independent variable significantly affects the dependent variable; (3) testing whether the mediating variable significantly change the influence of the independent variable on the dependent variable. If all three steps are satisfied, the mediation effect is confirmed. Results from M4 and M5 (Table 3) show that there is a significant positive correlation between PC and PIS (*β* = 0.367, *p* < 0.001), and H2 is proved. Furthermore, results from M6 show that both the first-order term (*β* = −0.384, *p* < 0.001) and the second-order term (*β* = −0.241, *p* < 0.001) of PIS are significantly negatively correlated with burnout, indicating that there is an inverted U-shaped relationship. H3 is proved. In addition, M7 shows that the first-order term and its second-order term of PIS are significantly negatively correlated with burnout. Compared with M2, the influence of PC on burnout changes from significant to insignificant. From this, it can be determined that PIS plays a mediating role between PC and burnout, and H4 is proved.

Furthermore, to avoid the potential distortion of the inverted U-shaped relationship in the pathway by Baron and Kenny’s three-step test and its inability to accurately reflect the mediating effect of the third variable, this study further tests the hypotheses using the MEDCURVE plug-in. This approach aims to effectively reduce Type I errors and ensure the robustness of the results (58). Specifically, the method uses bootstrap resampling to calculate the instantaneous indirect effect of the independent variable on the dependent variable through the mediator at different levels (M ± SD) of the independent variable. If the 95% confidence interval (95%CI) of the calculated effect does not include zero, the instantaneous mediating effect is deemed statistically significant. The results (see Table 4 part one) indicate that when the value of PC is relatively low *(XVAL = 2.7706)*, the mediating effect of PIS is negative [effect = −0.10, 95% CI (−0.1523,-0.0549)]. When the value of PC is at a medium level (XVAL = 3.7252), the mediating effect is negative [effect = −0.13, 95% CI (−0.2087, −0.0712)]. When the value of PC is relatively high (XVAL = 4.6800), the mediating effect is negative [effect = −0.17, 95% CI (−0.2653, −0.0824)]. The comprehensive analysis of the results unambiguously demonstrates that irrespective of the specific magnitude of PC, the estimated intervals of the mediating effect of PIS consistently exclude 0, thereby unequivocally attesting to the statistical significance of this mediating effect. Therefore, it can be concluded that PIS plays a mediating role in the curvilinear relationship between PC and burnout, reconfirming H4.

**Table 4:** The results of bootstrapping.

Pathway	Bianzhi	XVAL	θ	Lower CI	Upper CI
PC→PIS→ Burnout	Part one				
	Grassroots government employees	2.7706	−0.1001	−0.1523	−0.0549
		3.7252	−0.1336	−0.2087	−0.0712
		4.6800	−0.1670	−0.2653	−0.0824
	Part two				
	Binanei personnel	2.8778	−0.1089	−0.1745	−0.0609
		3.8031	−0.1361	−0.2300	−0.0729
		4.7284	−0.1633	−0.2869	−0.0815
	Bianwai personnel	2.4135	−0.0218	−0.1086	0.1074
		3.4236	−0.0629	−0.1944	0.0574
		4.4338	0.1040	−0.3344	0.0644

PC, Psychological contract; PIS, Perceived insider status.

### 4.4 The moderating effect of bianzhi

**Figure 3:**
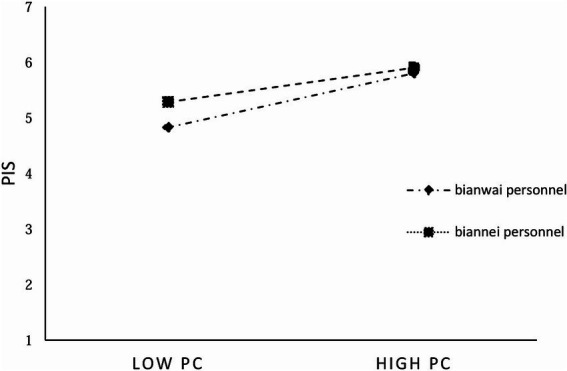
The moderating effect of bianzhi on PC and PIS.

According to M8, PC is significantly positively correlated with PIS (*β* = 0.514, *p* < 0.001), and the interaction term “PC × bianzhi” is significantly negatively correlated with PIS (*β* = −0.110, *p* < 0.05), indicating that bianzhi plays a negative moderating role. H5 is proved. Further, in order to better depict the moderating effect of bianzhi, a moderating effect diagram is made. It is indicated that, as shown in Figure 3, under different bianzhi categories, the extent of impact of PC on PIS of grassroots personnel varies. Specifically, PIS of bianwai personnel is more significantly influenced by PC.

### 4.5 Test of moderated mediating effect

This study uses bootstrapping to examine the moderated mediation effect model on an overall. The test results(see Table 4 part two) show that, for biannei personnel, when PC value is at a relatively low level [XVAL = 2.8778, 95% CI (−0.1745, −0.0609)], a moderate level [XVAL = 3.8031, 95% CI (−0.2300, −0.0729)], and a relatively high level [XVAL = 4.7284, 95% CI (−0.2869, −0.0815)], the instantaneous indirect impacts (θ values) of PC mediated by PIS invariably exclude 0 within the Bootstrapping 95% confidence interval. But with regard to the bianwai personnel, when the PC value is at a relativelt low level [XVAL = 2.87782.4135, 95% CI (−0.1086, 0.1074)], a moderate tier [XVAL = 3.4236, 95% CI (−0.1944, 0.0574)], and a relatively high level [XVAL = 4.4338, 95% CI (−0.3344, 0.0644)], the aforementioned instantaneous indirect effect (θ values) consistently includes zero in the bootstrapped 95% confidence interval. The empirical findings show that the mediating role of PIS is significant for biannei personnel but not for bianwai ones. Under the bianzhi influence, there’s a significant difference in the instantaneous indirect effect (θ values) of PIS between PC and burnout. The mediating effect of PIS among biannei personnel is stronger than bianwai ones. There is a moderated mediating effect between PC and burnout. H6 is proved.

The causes of this phenomenon can be further explored through the group differences between biannei and bianwai personnel. The fragility of bianwai personnel’ perception of PC is inherently deeply bound to the “job insecurity” prevalent in grassroots governance. In practical, bianwai personnel mostly establish employment relationships through short-term contracts, and there are institutional gaps between them and biannei personnel in terms of salary and benefits, job security, and promotion channels. This employment attribute dominated by transactional contract makes bianwai personnel’ perception of PC more directly affected by the stability of contract performance (such as contract renewal and salary payment). Once there is a gap in core guarantees, their PC perception will decline rapidly, and this decline is directly linked to burnout, without the need for transmission through the mediating path of PIS. In contrast, the antecedents of job burnout among biannei personnel exhibit the uniqueness of “excessive stability.” Although biannei personnel enjoy long-term job security, they face problems such as rigid career development and highly repetitive work, which are prone to continuously deplete their emotional resources, thereby triggering passive exhaustion and ultimately leading to burnout (70). At the same time, biannei personnel have stronger relational expectations of the organization (such as value recognition and career growth), and the gaps in PC will be further amplified through PIS. This also confirms the inherent logic that this variable has a significant mediating effect in the relationship between their PC and burnout.

It is worth further discussing that even though PIS does not exhibit a significant mediating effect among bianwai personnel, exploring this phenomenon still holds important significance. From the perspective of China’s grassroots governance practice, bianwai personnel have become an important subject in the performance of duties by grassroots governments, undertaking a large number of basic and operational tasks. Their level of burnout directly affects the quality of grassroots services and governance efficiency. Currently, bianwai personnel at the grassroots level in China generally face practical dilemmas such as “identity marginalization,” “inadequate protection,” and “limited development space.” The core of PIS lies in organizational identification and a sense of belonging, so testing and discussing the mediating effect of this variable essentially responds to the real psychological needs and living conditions of bianwai personnel.

From the perspective of practical value, first, this result provides a precise target for Chinese grassroots governments to optimize the management of bianwai personnel. The insignificant mediating effect of PIS indicates that simply fostering PIS is difficult to fundamentally alleviate their burnout. Instead, it is necessary to focus on transactional contract elements such as fair compensation and clear performance incentives to meet their core demands and avoid the ineffective investment of management resources. Second, this phenomenon reflects the “psychological barrier” brought about by bianzhi, providing empirical evidence for promoting the reform of Chinese grassroots human resource system. The difference in the mediating effect of PIS confirms that the psychological alienation caused by bianzhi differences is a core pain point in the management of bianwai personnel. Future reforms need to break down bianzhi barriers through institutional design, such as endowing equal training opportunities and promotion channels, thereby fundamentally alleviating the burnout of bianwai personnel. Third, this discussion helps China’s grassroots governments clarify the management differences between the two groups and achieve classified governance, so as to maximize the work engagement of both groups and lay a foundation for realizing Chinese-style modernization.

## 5 Discussion

### 5.1 Research findings

This study has yielded several significant findings. Firstly, this study empirically verifies a non-linear inverted U-shaped relationship between PC and burnout among Chinese grassroots government employees, breaking through the generally recognized cognitive framework of a linear correlation in existing research. The study finds that the relationship between PC and burnout is a non-linear inverted U-shaped relationship. Specifically, when there is a significant gap between the actual perception of grassroots government employees and their expectations, negative disconfirmation is formed. At this time, the level of job burnout of grassroots government employees will not decrease with the improvement of PC, but it will increase due to unmet expectations. In contrast, when the actual perception of grassroots government employees is close to or exceeds their expectations, positive disconfirmation is formed, which drives a decrease in burnout. This conclusion, on the one hand, expands and deepens the understanding of EDT and SRT. Penning de Vries and Knies (59) confirmed through two experiments that employees’ perceived disappointment with supervisory support significantly impairs their job satisfaction. Essentially, this impact stems from the perceived gap between expectations and reality, which aligns with the core logic revealed in this study, that negative disconfirmation induces burnout. Together, these findings validate the applicability of EDT in public sector contexts.

Job burnout is a comprehensive reaction characterized by physical and mental exhaustion formed, when individuals perceive that the work requirements and personal investment do not match the organizational return (29). When there is a negative inconsistency between an individual’s expectation of the organization fulfilling its commitments or responsibilities and the actual feeling, employees often develop dissatisfaction. At the same time, they will also respond with strong negative attitudes and behaviors, such as negative emotions and burnout behaviors that deviate from organizational interests (60), so as to achieve the purpose of reducing the gap between their own efforts and rewards. This is consistent with the viewpoints in EDT that “individual satisfaction is based on the relative magnitude of expectations and actual perception,” and in SRT that “individuals will make adaptive behaviors in combination with the organizational environment.” And this finding provides empirical evidence for expanding the application scenario of EDT to the field of organizational behavior. On the other hand, it provides empirical test evidence for the view in COR that “the degree of influence of resource depletion and resource acquisition is not completely consistent,” and indirectly illustrates the importance of organizational PCs management. The negative consequences brought about by PCB may be far greater than the positive impacts brought about by PCF.

Secondly, this study reveals the mediating role of PIS between PC and burnout, while clarifying the nonlinear characteristics of this mediating pathway. Based on the “PC-PIS-burnout” pathway, this research finds that PIS plays a mediating role in this relationship. Specifically, in the latter segment of this influence pathway, an inverted U-shaped relationship exists between PIS and burnout. That is, when grassroots government employees hold high expectations for insider status, a significant gap between their actual perception and expectations will form negative disconfirmation. Under such circumstances, the burnout level of grassroots government employees increases as their PIS improves. This discovery holds two important implications. On the one side, combined with EDT and SRT, it reveals the inverted U-shaped mediating role of PIS, and builds a bridge for explaining the internal mechanism between the gap generated by an individual’s “expectation actual perception” and behavioral response. On the other side, it expands the antecedent research on burnout of grassroots government employees, indicating that PIS of Chinese grassroots government employees is also an important factor affecting their burnout. At the same time, it also verifies the view in the perspective of self-control resources that psychological resources can effectively alleviate irrational behaviors due to the loss of self-control resources (61, 62).

Finally, it has been confirmed that bianzhi moderates the process through which PC influences PIS and moderates the mediating effect of PIS. This study indicates that bianzhi weakens the relationship between PC and PIS. That is, under any PC state (whether PCF or PCB), the level of PIS of bianwai personnel is usually lower than that of biannei personnel. When the grassroots government employees are in a PCB state, the gap in PIS between bianewai personnel and biannei personnel will widen significantly, and PIS of bianwai personnel will be significantly lower than that of biannei personnel. This conclusion is highly consistent with the findings of Xia et al. (63) on temporary employees in Chinese local governments. Through an empirical analysis of 820 government temporary employees (bianwai personnel). This study found that due to the temporary nature of their status, bianwai personnel’ level of organizational commitment is significantly lower than that of biannei personnel. Meanwhile, organizational commitment is strongly positively correlated with PIS, indicating that bianwai personnel’s PIS is more dependent on the level of PC, and their PIS is more susceptible to the degree of organizational contract fulfillment. More importantly, through the moderated nonlinear mediating model constructed in this study, it is found that the mediating effect of PIS is only significant among the biannei group but not significant among the bianwai group. It not only clearly distinguishes the burnout formation mechanisms between the two groups but also addresses the limitation of previous studies that overlooked the uniqueness of the “bianzhi boundary” in the public sector, thereby providing a more precise analytical framework for comprehensively explaining the influencing factors and action paths of burnout among Chinese grassroots government employees.

### 5.2 Practical implications

The aforementioned theoretical findings deepen the understanding of the complex relationships between grassroots government employees’ PC, PIS, bianzhi and job burnout, at the same time, it provides clear operational logic for addressing the practical dilemma of “high incidence of government employee burnout and insufficient governance effectiveness” in current Chinese grassroots governance. These findings need to be transformed into operable practical strategies to effectively respond to the psychological demands and work predicaments of grassroots government employees, and practically improve their job burnout situation. In this regard, integrating the governance scenarios and personnel management characteristics of Chinese grassroots governments, and drawing on the research findings of psychological contracts in similar public service-oriented occupations, this study proposes specific practical strategies from three dimensions: psychological contract management, stimulation of perceived insider status, and weakening of identity differences arising from bianzhi. It aims to provide an action plan for effectively alleviating the job burnout of Chinese grassroots government employees, optimizing grassroots human resource management, and enhancing the effectiveness of Chinese grassroots governance.

To begin with, the organizations should prioritize strengthening the PC management of grassroots government employees. This can be achieved through multiple means. On the one hand, establishing a relatively equitable salary and treatment system is essential to fulfill transactional contracts and optimize the balance between work requirements and salary and welfare. Studies have shown that transactional contract, centered on economic benefits and job security, constitutes a core dimension of PC (64). Fair compensation can effectively reduce individuals’ perceived PCB and significantly enhance their sense of organizational belonging and satisfaction (65). In response to this, organizations should appropriately increase the proportion of performance appraisal rewards and adopt flexible compensation methods such as agreement-based wage systems or annual salary systems, so as to maximize the balance between work requirements and compensation and benefits. On the other hand, improving the promotion assessment system for grassroots government employees is crucial for fulfilling relational contracts. This may involve implementing a dynamic tracking mechanism for promotion results to ensure their effectiveness and establishing a management paradigm where capable individuals are promoted while incompetent ones are demoted or even removed. Additionally, for bianwai personnel, creating a dedicated promotion mechanism can effectively stimulate their work motivation. Equally important is assisting employees in setting appropriate work expectations to avoid “expectation misalignment” between individuals and organizations. At the same time, organizations should be vigilant against the occurrence of the “medium trap” in PCs.

Furthermore, organizations should focus on enhancing PIS among grassroots government employees. One approach is to modify leadership management styles to foster employees’ sense of gratitude and reciprocity. Alternatively, fully empowering employees and allowing them to make independent decisions can also stimulate their sense of ownership, thereby elevating their PIS. Evidence suggests that involving employees in management communication and decision-making can to a certain extent fulfill their PC and commitment needs, thereby enhancing their work motivation (66). However, it should be noted that, considering the finding of an inverted U-shaped relationship between PIS and burnout, organizations need to be cautious about falling into the “medium level trap” of PIS in practice and prevent the emergence of high job burnout due to individuals being in an intermediate state of PIS.

Finally, organizations should strive to reduce the identity differences caused by bianzhi categories. On one hand, foster a positive organizational ecology and cultural atmosphere. Zhang (67) pointed out that inadequate fulfillment of organizational responsibilities will exacerbate employees’ perceived PCB, and endowing employees with due social status and respect can significantly reduce their level of burnout. Therefore, organizations should break down the “barriers” caused by bianzhi differences and narrow the gap in PIS between biannei and bianwai personel by encouraging bianwai personnel to equally participate in various organizational activities, providing them with certain collegial and organizational care, and reducing the sense of alienation in language and actions. On the other handthe government should continue to actively promote bianzhi management reform and explore diversified personnel management models, such as bianzhi management mechanism of extra-bianzhi (68), flexible adjustment of bianzhi management (69), and innovative classification methods for bianzhi (41).

### 5.3 Research limitations and outlook

This study adopted a combined method of multistage stratified sampling and simple random sampling to determine the sample. It not only covers different types of grassroots departments but also includes diverse individual characteristics, effectively reducing selection bias and laying a solid foundation for the generalizability of the research results. Therefore, the conclusions of this study can be generalized to Chinese grassroots governments with similar governance scenarios and personnel characteristics.

Despite the valuable insights obtained from this study, certain limitations exist. Primarily, due to resource constraints, the sample was unable to cover grassroots government employees from all regions of China, thereby necessitating an expansion of the questionnaire’s breadth and scope in future research. Additionally, the current study lacks a detailed analysis of the relationships among the various subdivided dimensions of PC, PIS, and burnout. Given that there may be significant differences in the influence mechanisms among these dimensions, future research could conduct in-depth investigations into the micro-influence mechanisms. Finally, the current study did not further classify and discuss the bianzhi types of grassroots biannei personnel. There might be variations in the moderating effects of administrative bianzhi and subsidiary bianzhi on the relationship between PC and PIS. Moreover, the influence of jiebian, a common phenomenon in grassroots governments where a superior unit borrows personnels from a subordinate one, has not been considered. Since borrowed personnels may experience reduced work efficiency, weakened PIS, and burnout due to salary differences among hierarchical administrative units, future research could classify grassroots government employees based on bianzhi types and employment methods (i.e., jiebian) to obtain more profound and accurate research results.

In conclusion, this study has made significant contributions to understanding the relationships among PC, PIS, bianzhi, and burnout among Chinese grassroots government employees. However, further research is warranted to address the identified limitations and expand the knowledge in this area.

## 6 Conclusion

This study uses survey data from 527 grassroots government employees in China. Based on EDT and SRT, a moderated mediation effect model is constructed to explore the nonlinear relationship between PC and burnout of grassroots government employees in China, as well as the mediating role of PIS and the moderating role of bianzhi. The research results show that there is an inverted U-shaped relationship between PC and burnout, an inverted U-shaped relationship between PIS and burnout of grassroots government employees in China. PC has an inverted U-shaped mediating effect on burnout through PIS. Bianzhi plays a moderating role in PC and PIS. That is, under any PC state (whether PCB or PCF), the level of PIS of biannei personnel is significantly higher than that of bianwai personnel. The nonlinear mediating mechanism of PC affecting burnout through PIS is moderated by bianzhi. That is, the mediating effect of PIS is significant among biannei personnel but not significant among bianwai personnel.

## Data Availability

The raw data supporting the conclusions of this article will be made available by the authors, without undue reservation.
